# Effect of Liraglutide on Intermittent Hypoxia‐Induced Metabolic Dysfunction: From Bench to Bedside

**DOI:** 10.1111/jsr.70152

**Published:** 2025-07-28

**Authors:** Cliona O'Donnell, Ailbhe King, Guillaume Vial, Emily O'Neill, Shane Crilly, Jonathan D. Dodd, David J. Murphy, Elise Belaidi, Jean‐Louis Pepin, Claire Arnaud, Donal O'Shea, Silke Ryan

**Affiliations:** ^1^ Pulmonary and Sleep Disorders Unit St. Vincent's University Hospital Dublin Ireland; ^2^ School of Medicine University College Dublin Ireland; ^3^ University Grenoble‐Alpes, Laboratoire HP2, INSERM U1300 Grenoble France; ^4^ Department of Radiology St. Vincent's University Hospital Dublin Ireland; ^5^ Department of Endocrinology St. Vincent's University Hospital Dublin Ireland

**Keywords:** continuous positive airway pressure, GLP‐1 receptor agonists, intermittent hypoxia, Liraglutide, obstructive sleep apnoea, pharmacological weight loss

## Abstract

Intermittent hypoxia (IH)‐mediated adipose tissue inflammation with M1 macrophage polarisation plays a key role in the pathogenesis of metabolic diseases in obstructive sleep apnoea (OSA). Effective treatment strategies are so far lacking. Here, we hypothesised that a glucagon‐like peptide (GLP)‐1 (Liraglutide)‐based weight loss regimen improves IH‐induced metabolic perturbations. To test the hypothesis, we employed a comprehensive translational approach consisting of an innovative IH system for cell cultures, a murine IH model of diet‐induced obese mice and a proof‐of‐concept randomised‐controlled study in OSA (NCT04186494). Liraglutide significantly attenuated IH‐mediated pro‐inflammatory polarisation of bone marrow‐derived murine macrophages in cell culture. However, this did not translate into improved IH‐induced insulin resistance in C57Bl/6 mice fed on a high‐fat diet despite significant weight loss. In OSA subjects without diabetes (*n* = 30, 50 ± 7 years, 80% males, apnoea–hypopnoea index [AHI] 50 ± 19/h, body mass index [BMI] 35.0 ± 3 kg/m^2^), Liraglutide in contrast to CPAP over 24 weeks led to improvement in insulin sensitivity (mean difference 1.91 ± 1.46 vs. −1.02 ± 2.75, *p* = 0.03) in correlation with reduction in anthropometric measures and visceral adipose tissue volume. However, in conjunction with its limited effect on OSA parameters, the combination of Liraglutide with CPAP therapy appeared superior to Liraglutide alone for improvement of other glycaemic parameters such as fasting glucose, glucose tolerance, or HbA1c. In summary, while Liraglutide is effective in mediating weight loss, a lack of improvement in IH‐triggered metabolic dysfunction does not support its role as monotherapy for metabolic diseases in OSA.

## 1 Introduction

Obstructive sleep apnoea (OSA) is an independent risk factor for metabolic disorders such as insulin resistance (IR) and type 2 diabetes (T2D) (Ip et al. 2002; Kent et al. 2014, 2015). Intermittent hypoxia (IH) is a key pathophysiological driver of metabolic dysfunction in OSA, mediating its action, at least in part, through promoting a pro‐inflammatory, metabolically dysfunctional phenotype of the visceral adipose tissue (AT) (Murphy et al. 2017; Ryan et al. 2019).

The benefit of continuous positive airway pressure (CPAP) therapy on metabolic diseases in OSA is uncertain (Jullian‐Desayes et al. 2015; Labarca et al. 2018) and similarly in rodent models, cessation of IH only results in partial resolution (Gileles‐Hillel et al. 2017; Polak et al. 2013). In a landmark study by Chirinos et al. (2014), weight loss was superior to CPAP in improving metabolic function, but there appeared to be a potentially synergistic effect with both treatment modalities combined. Weight loss, however, is difficult to achieve and maintain with conventional measures alone. The area of pharmacologically mediated weight loss has expanded rapidly in recent years and has led to a critical paradigm shift in the understanding of obesity as a holistic disease. Glucagon‐like‐peptide (GLP)‐1 receptor agonists such as Liraglutide and Semaglutide lead to weight reduction of up to 15% (Pi‐Sunyer et al. 2015; Wilding et al. 2021) and this benefit is succeeded even further by some dual‐ and tri‐receptor agonists (Jastreboff et al. 2022, 2023). The proposed mechanisms of actions of GLP‐1 agonists include delayed gastric emptying, increased satiety and appetite suppression (Drucker 2022). In addition, accumulating evidence supports their direct effect on the AT through attenuation of inflammation and ‘browning’ with downstream effects of improved metabolic function (Beiroa et al. 2014; Lee et al. 2012; Lynch et al. 2016).

In line with the important role of obesity as a modifiable factor in the pathophysiology of OSA, GLP‐1 analogues have gained fast increasing attention in the field. In particular, the recent Surmount‐OSA trial comparing the dual receptor agonist Tirzepatide to placebo in subjects with OSA and obesity promoted this treatment as a potential management option for a large proportion of OSA patients (Malhotra et al. 2024). However, most weight loss studies have focused primarily on changes in OSA parameters, and the effect of GLP‐1‐based pharmacotherapy on OSA‐related metabolic outcomes remains poorly explored. We have recently reported that Liraglutide‐based weight loss was inferior to CPAP therapy in its effect on early vascular disease processes (O'Donnell et al. 2024). However, given the central role of AT dysfunction in metabolic diseases, its action may be primarily targeted towards such consequences. In support of this hypothesis, the SCALE Sleep Apnoea trial reported improvements in fasting glucose and HbA1c through Liraglutide‐mediated weight loss, but the intervention was explored only in subjects unwilling or unable to use CPAP, and its effect on IR was not assessed (Blackman et al. 2016).

Here, we hypothesised that the GLP‐1 analogue Liraglutide improves insulin sensitivity and other glucose metabolic parameters in OSA. We explored this hypothesis using a comprehensive translational approach ranging from our state‐of‐the‐art IH system for cell cultures over our extensively validated murine model to a proof‐of‐concept randomised controlled study in OSA involving detailed characterisation of anthropometrics, insulin sensitivity and other metabolic indices.

## 2 Methods

### 2.1 Cell Culture and in Vitro Model of IH

Bone marrow derived macrophages (BMDMs) were isolated from male C57BL/6 mice and grown on a semi‐permeable membrane (Lumox, Sarstedt, Nuermbrecht, Germany) as previously described (Fitzpatrick et al. 2021). After 7 days in culture, mature BMDMs were treated with 250 nM Liraglutide or with phosphate‐buffered saline (PBS) before exposure to the IH or control protocol as previously reported (Murphy et al. 2017). Briefly, the IH protocol consists of alternating cycles of 40 s of atmospheric 16% O_2_/5% CO_2_/balance N_2_ and 40 s of 3% O_2_/5% CO_2_/balance N_2_ applied for 8 h/day for 2 consecutive days resulting in cellular pO_2_ levels similar to IH experienced by OSA subjects (between ~5 and 12 kPa). Cells exposed to intermittent air (IA) as control are treated with 16% O_2_/5% CO_2_/balance N_2_ at the same flow and time rates. Temperature during the whole protocol is kept constant at 37°C. Upon completion of the protocol, RNA was isolated for real‐time PCR as previously described (Fitzpatrick et al. 2021). Supernatants were stored at −80°C and interleukin‐6 (IL‐6) ELISA was performed as per manufacturer's instructions (R&D Systems).

### 2.2 Mice

Forty 15‐week‐old male wild‐type C57BL/6 mice were commenced on a 60% high‐fat diet (HFD) (D12492, Research Diets Inc.). Following a run‐in time of 1 week, mice were randomised to 6 weeks of IH or IA control as previously described (Murphy et al. 2017) with daily subcutaneous administration of Liraglutide (incremental increase to 300 μg/kg) or PBS as control. Weight and food intake were monitored daily.

Upon completion of the protocol, fasting glucose and insulin were measured and an intraperitoneal insulin tolerance test (ITT) was performed as previously described (Poulain et al. 2014). Fifteen minutes prior to sacrifice, mice were injected with either insulin (0.5 U/kg) or NaCl for in vivo insulin stimulation. Epididymal adipose tissue (eWAT) and liver were harvested and stored at −80°C for further analysis. For western blotting, whole tissue protein extractions were separated by SDS‐PAGE, transferred to nitrocellulose membranes, and immunoblotted. Primary antibodies against Phospho‐Akt (Ser43, Cell Signalling 9271S), total Akt (Cell Signalling 9272S) and β‐actin (Merck A5441‐100UL) were used, as well as species‐specific HRP‐conjugated secondary antibodies. Image J software was applied to quantify the western blot signals. The study was approved by the Institutional Animal Care and Use Committee, and mice were maintained in accordance with the European Convention for the Protection of Vertebrate Animals used for Experimental and other Scientific Purposes.

### 2.3 Human Study

We recently reported on the design, protocol with detailed inclusion and exclusion criteria, and baseline characteristics of this proof‐of‐concept randomised controlled trial (O'Donnell et al. 2024). In brief, 30 non‐diabetic subjects aged 18–60 years with newly diagnosed moderate to severe OSA (apnoea–hypopnoea index [AHI] > 15/h) and with a body mass index (BMI) between 30 and 40 kg/m^2^ were recruited from our Sleep Laboratory and randomised to one of three arms: CPAP therapy alone, a Liraglutide‐based weight loss regimen alone or both in combination, for 24 weeks. The primary outcome of the study was to assess the impact of those treatment strategies on IR measured via HOMA‐IR. Following overnight polysomnography (Murphy et al. 2017), fasting blood was drawn for metabolic markers including HbA1c, glucose and insulin before undergoing oral glucose tolerance testing (75 g glucose load [Rapilose, Galen, UK]). All measurements were performed at baseline and at 24 weeks. The study was approved by the Ethics Committee of St. Vincent's University Hospital, registered with ClinicalTrials.gov (NCT04186494) and participants provided written informed consent.

### 2.4 Measurements of AT Volume by 18F‐FDG PET‐CT

AT volume measurements were performed on the non‐contrast CT component of the PET‐CTs using Syngo Via post‐processing software (Siemens Healthineers, Erlangen, Germany) using a previously validated technique (Lee et al. 2015). Measurements were performed on the axial CT images with a 1 mm slice thickness at the level of L3–L4 intervertebral disk space by an expert radiologist (D.J.M.), blinded to patient treatment groups. The total fat volume (TFV) was measured by auto‐contouring the outer skin surface with manual editing, where appropriate. The cross‐sectional fat volume (in cm^3^) within the contours was then measured by Hounsfield unit threshold segmentation (range −40 to −200). The visceral fat volume (VFV) was then measured by repeating these steps with the contours drawn immediately deep to the innermost surface of the abdominal wall musculature. The subcutaneous fat volume (SFV) was calculated by subtracting the VFV from the TFV.

### 2.5 Statistical Analysis

All data are expressed as mean ± SD or mean ± SEM as indicated. Group comparisons were performed using one‐way ANOVA followed by Bonferroni post hoc comparison. Within‐group tests were performed using paired *t* tests. To identify the effect of body weight and pharmacological intervention in the murine study, an analysis of covariance (ANCOVA) model using body weight as a covariate, treatment as a fixed factor, and glucose as the dependent variable was used.

The human study element of our project was an exploratory, proof‐of‐concept study with the purpose of aiding the planning of future larger RCTs. Therefore, all statistical analysis presented is for the purpose of exploring signals in treatment differences between groups. Depending on distribution, Pearson's or Spearman's Rho analysis was used to assess for univariate correlation between variables. To assess the possible confounding effect of weight on metabolic parameters in the mice experiment, a general linear model to assess for ANCOVA was utilised. To identify potential independent predictors of the change in insulin sensitivity in OSA subjects, we employed a stepwise linear regression model with change in HOMA‐IR as the dependent variable and change in demographic, anthropometric, AT volumes and PSG variables as covariates. A *p* value of < 0.05 was considered statistically significant. All statistical analysis was carried out using IBM SPSS Statistics Version 27.

## 3 Results

### 3.1 Liraglutide Attenuates IH‐Mediated Pro‐Inflammatory Polarisation of Cultured Primary Macrophages

**FIGURE 1:**
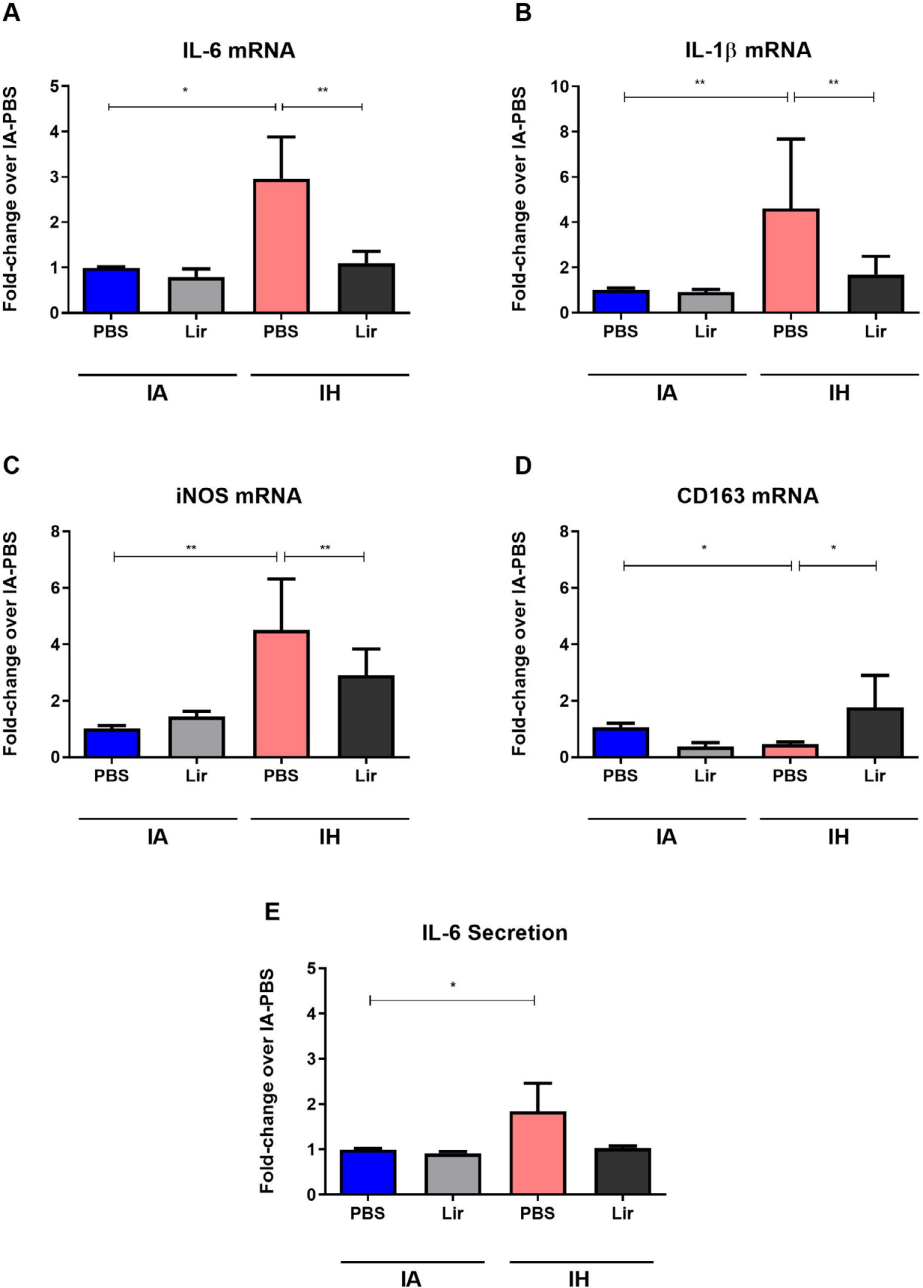
Liraglutide attenuates intermittent hypoxia (IH)‐mediated pro‐inflammatory macrophage polarisation in vitro. Bone‐marrow derived macrophages (BMDMs) were obtained from wild‐type C57BL/6 male mice and exposed to IH or intermittent air (IA) for 8 h on 2 consecutive days in the presence of 250 nM Liraglutide or PBS. mRNA was extracted and reversely transcribed and real‐time PCR was performed to assess for the expression of the pro‐inflammatory markers interleukin (IL)‐6 (A), IL‐1β (B), inducible nitric oxide synthase (iNOS) (C) and the M2 macrophage marker CD163 (D). Following the experiments supernatants were collected and concentration of IL‐6 was measured by ELISA (E). Data are presented as mean ± SEM, *p < 0.05, **p < 0.01, *n* = 5.

We have previously demonstrated that IH polarises primary murine bone‐marrow‐derived macrophages (BMDM's) towards a pro‐inflammatory M1 phenotype in vitro (Fitzpatrick et al. 2021). Here, we evaluated the effect of pharmacological intervention with the GLP‐1 analogue Liraglutide on this response. As demonstrated in Figure 1, Liraglutide significantly attenuated the IH‐induced mRNA expression of the pro‐inflammatory markers IL‐6, IL‐1β and inducible nitric oxide synthase (iNOS) (Figure 1A–C). In contrast, the IH‐reduced expression of the M2 anti‐inflammatory marker CD163 was restored by Liraglutide (Figure 1D). Liraglutide also decreased IH‐induced IL‐6 protein secretion; however, this did not reach statistical significance (Figure 1E).

Taken together, Liraglutide counteracts the IH‐induced pro‐inflammatory macrophage polarisation in vitro.

### 3.2 Liraglutide Leads to Significant Weight Loss in C57BL/6 Male Mice

**FIGURE 2:**
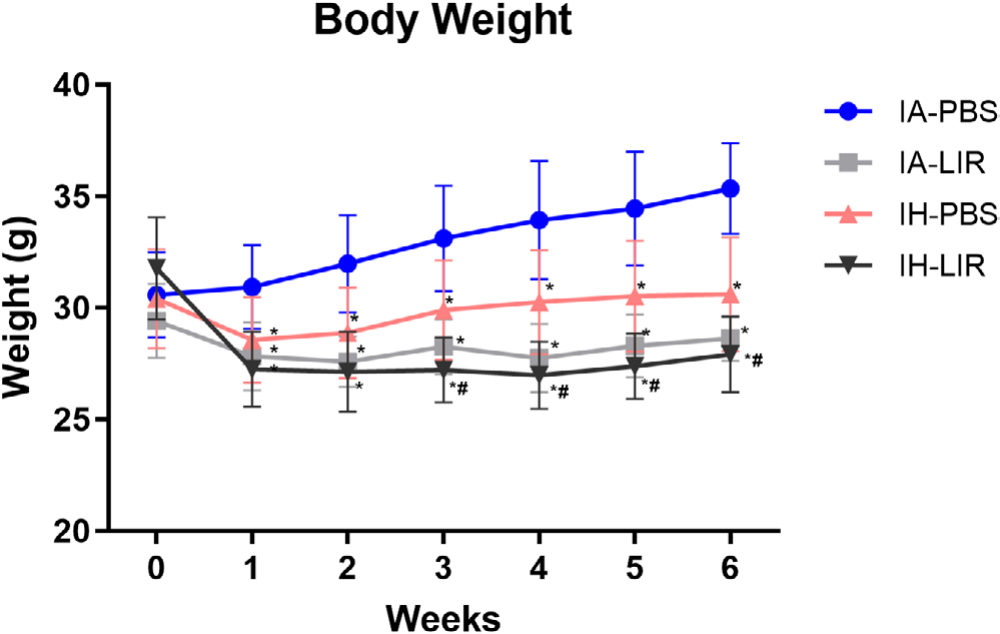
Liraglutide leads to inhibition of weight gain in mice fed on a high‐fat diet. C57BL/6 male mice were fed a 60% high‐fat diet. Following a run‐in time of 1 week, they were randomised to intermittent air (IA) or intermittent hypoxia (IH) treatments with daily subcutaneous Liraglutide or phosphate‐buffered saline (PBS) administration. Body weight was monitored weekly. Data are presented as mean ± SEM for *n* = 9–10 per group. *compared to IA‐PBS, ^#^compared to IH‐PBS. */^#^ *p* < 0.05.

We next aimed to evaluate whether those effects translate into improvement of IH‐related IR in C57BL/6 wild‐type male mice fed on a high‐fat westernised diet. First, we assessed the impact of GLP‐1 analogue treatment on body weight. Mice were exposed to 6 weeks of IH or IA as a control condition, treated with daily subcutaneous injections of Liraglutide or PBS, and were weighed daily. As expected in rodent studies, IH‐treated animals gained significantly less weight than controls, but in both conditions, Liraglutide led to a further significant weight loss (Figure 2) in conjunction with reduced food intake, and this was also associated with reduction in the AT weights (Table 1).

**TABLE 1:** Final body weight, adipose tissue weights and overall food intake.

	IA‐PBS	IA‐LIR	IH‐PBS	IH‐LIR
Final body weight (g)	35.3 ± 2.03	28.6 ± 1.03*	30.6 ± 2.55*	27.9 ± 1.71^#^ *
Overall food intake (g)	18.7 ± 1.06	15.71 ± 1.65*	14.9 ± 1.22*	14.7 ± 1.93*
eWAT weight (g)	1.6 ± 0.65	0.48 ± 0.24*	0.84 ± 0.47*	0.31 ± 0.07^#^ *
scWAT weight (g)	0.8 ± 0.29	0.29 ± 0.12*	0.48 ± 0.30*	0.25 ± 0.05^#^ *

*Note*: Data represent mean ± SD.

Abbreviations: eWAT = epididymal white adipose tissue, scWAT = subcutaneous white adipose tissue.

* *p* < 0.05 versus IA‐PBS.

^#^ *p* < 0.05 versus IH‐PBS.

Thus, Liraglutide leads to effective weight loss in C57BL/6 mice.

### 3.3 Liraglutide Improves Fasting Glucose but Does Not Impact on IH‐Mediated Systemic and AT Insulin Resistance in C57BL/6 Mice

**FIGURE 3:**
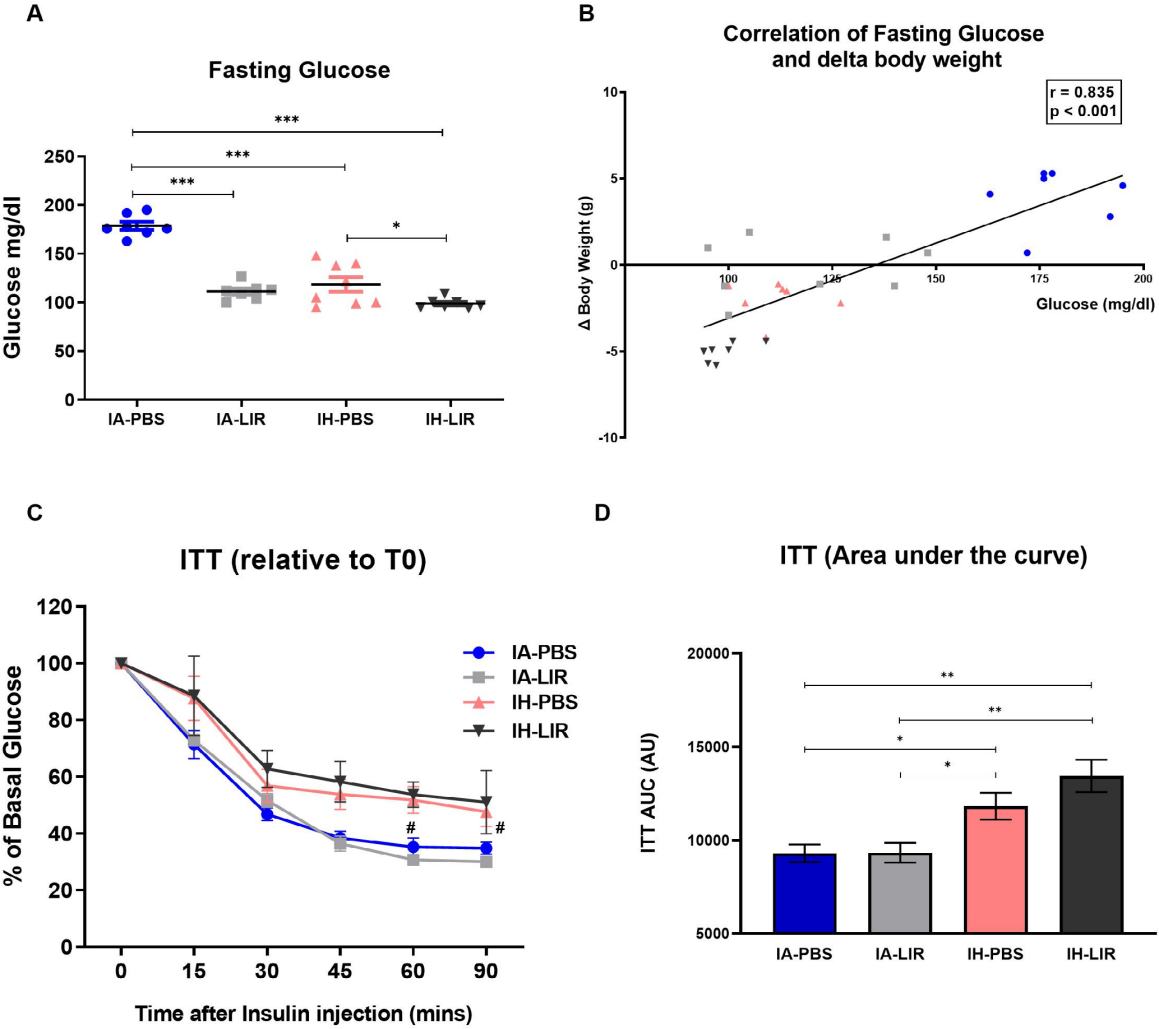
IH decreases insulin sensitivity, without modifying effects of Liraglutide. Fasting blood glucose was measured at the end of the protocol (*n* = 7–8 per group) (A) and a bivariate correlation analysis was performed to assess the relationship between fasting glucose and change in body weight (B). To evaluate for insulin sensitivity, an intraperitoneal insulin tolerance test (ITT) was performed following 6 h of fasting. The ITT is represented as time course relative to glucose levels at baseline (C) (*n* = 9–10 per group) and as area under the curve (AUC) (D). Data represent mean ± SEM. *p < 0.05, **p < 0.01, ***p < 0.001, ^#^IH versus IA=*p* < 0.05.

In conjunction with the highest body weight of mice treated with IA and PBS, fasting glucose levels were also highest in this group (Figure 3A) and there was a significant correlation between fasting glucose and change in body weight from baseline (Figure 3B).

Liraglutide significantly reduced fasting glucose in both IA and IH conditions, and using an ANCOVA with body weight as a covariate, Liraglutide treatment remained a significant predictor of lower glucose (*p* < 0.001). However, Liraglutide had no effect on fasting insulin levels in IH‐treated mice (PBS vs. Liraglutide: 1.29 ± 0.60 vs. 0.91 ± 0.20 ng/mL, *p* = 0.138).

We have previously demonstrated that IH contributes to IR in C57BL/6 wild‐type mice, independent of the presence of obesity (Murphy et al. 2017) and this adverse effect was reproduced in this study as evaluated by ITT. However, Liraglutide treatment did not alter this effect in either IA or IH conditions (Figure 3C,D).

**FIGURE 4:**
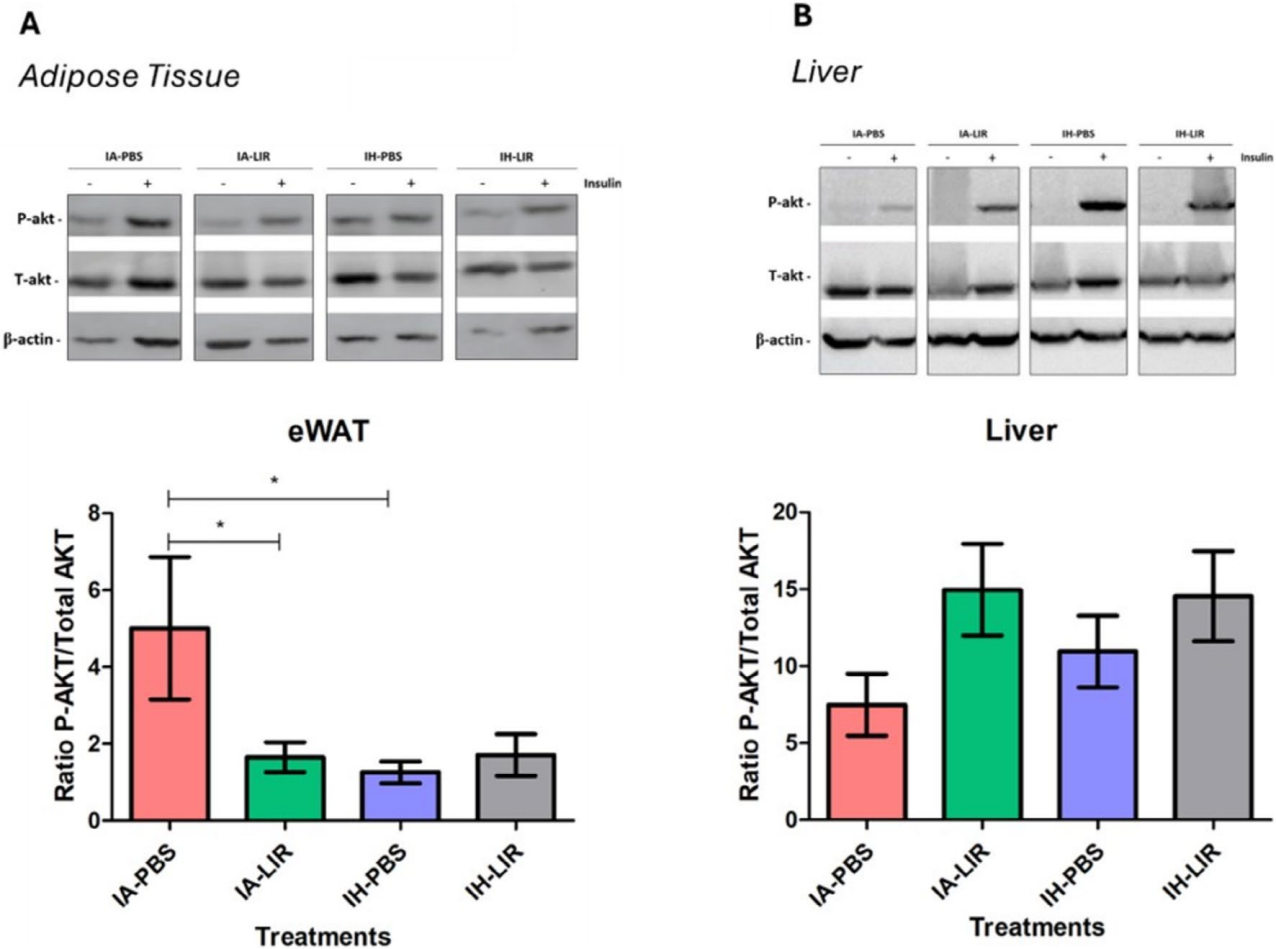
Intermittent hypoxia (IH)‐induced visceral adipose tissue insulin resistance remains unaltered by Liraglutide. 15 min prior to sacrifice, mice were injected with either NaCl or 0.5 U/kg insulin. Visceral adipose tissue (A) and liver (B) insulin sensitivity were measured via P‐AKT/AKT ratio in extracts using western blotting (*n* = 4–6). Presented is a representative blot and densitometry graphs (mean ± SEM) which were quantified using ImageJ software. *p < 0.05.

To assess for organ‐specific IR, we evaluated Ser43‐phosphorylation of Akt, a major player in the insulin signalling cascade, in eWAT and liver following insulin injection prior to sacrifice. As shown in Figure 4A, IH significantly reduced insulin‐dependent Akt phosphorylation in comparison to control treatment. However, Liraglutide intervention did not reverse or attenuate those effects and had no beneficial effect in IA. Neither IH treatment nor Liraglutide administration had a significant effect on insulin signalling in the liver (Figure 4B).

Thus, although Liraglutide led to significant weight loss associated with improvement in fasting glucose, the IH‐induced systemic and adipose‐tissue IR remained unaffected.

### 3.4 Liraglutide Leads to Significant Weight Loss in Subjects With OSA

**FIGURE 5:**
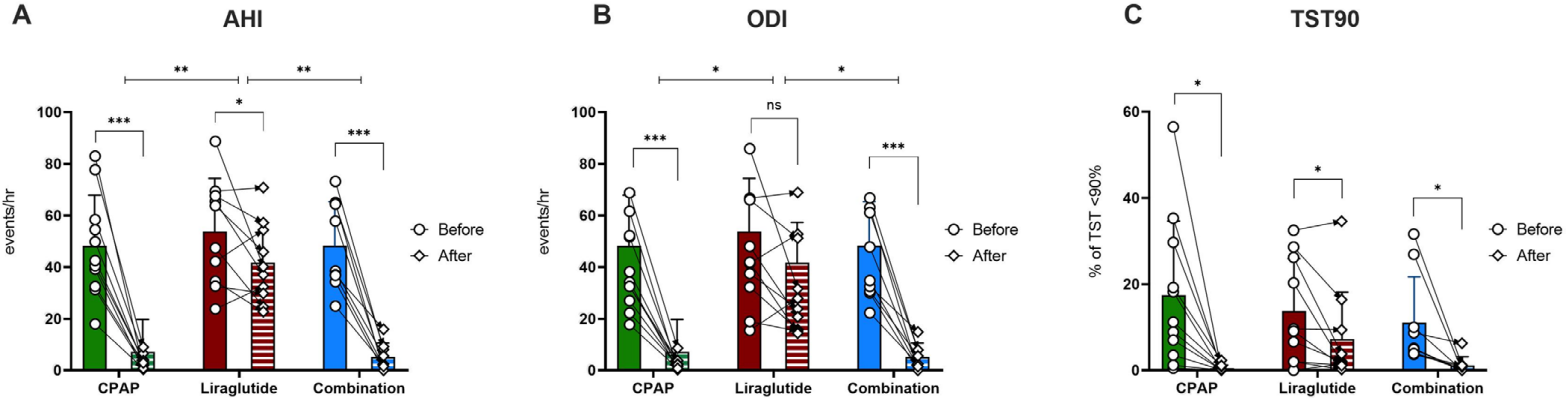
CPAP treatment is more effective in improving AHI and markers of intermittent hypoxia than Liraglutide. Change in apnoea/hypopnoea index (AHI) (A), oxygen desaturation index (ODI) (B) and (C) percent of total sleep time spent at oxygen saturation below 90% (TST90) in the three treatment groups. Data represent mean ± SD and individual point‐to‐point changes, *p < 0.05, **p < 0.01, ***p < 0.001.

Having established that Liraglutide, despite weight loss, does not improve IH‐induced IR in mice fed on a HFD, we next aimed to gain insight into the role of this pharmacological approach on metabolic function in subjects with OSA and obesity. We recently described the design and progress of this proof‐of‐concept study comparing a Liraglutide‐based weight loss regimen alone, CPAP therapy alone, or both in combination for 24 weeks (O'Donnell et al. 2024). Briefly, groups were similar in baseline demographic and sleep study parameters (Table 2). Mean CPAP adherence in the CPAP and combination groups was 5.8 ± 1.4 and 4.7 ± 1.8 h per night, respectively, and adherence to Liraglutide was also good, as reported by the subjects and by monitoring the contents of the pre‐filled pens. As previously reported (O'Donnell et al. 2024), CPAP alone or in combination resulted in effective treatment of OSA and abolishment of IH (Figure 5). Liraglutide was also accompanied by significant improvement in all OSA‐related parameters, but to a significantly lesser degree than in the other two groups (Figure 5). Subjective sleepiness evaluated by the Epworth Sleepiness Scale (ESS) trended to improve in all groups, but the difference only reached statistical significance with CPAP treatment (CPAP: from 8 ± 5 to 4 ± 2, *p* = 0.015, Liraglutide: from 8 ± 6 to 6 ± 4, combination: from 10 ± 5 to 8 ± 5).

**TABLE 2:** Baseline characteristics of the study population using the final analysis allocation.

	Total	Liraglutide (*n* = 10)	CPAP (*n* = 11)	Combination (*n* = 9)	*p*
Female	6 (20)	2 (20)	1 (9)	3 (33)	0.403
Age (years)	50 ± 7	50 ± 10	49 ± 8	51 ± 4	0.919
BMI (kg/m^2^)	34.9 ± 3.2	35.1 ± 3.0	35.0 ± 3.5	34.4 ± 3.2	0.878
*Smoking status*					
Current smoker	3 (10)	0 (0)	2 (18)	1 (11)	0.379
Ex‐smoker	16 (53)	5 (50)	6 (55)	5 (56)	0.966
Never‐smoker	11 (37)	5 (50)	3 (27)	3 (33)	0.542
Epworth Sleepiness Scale	9 ± 5	8 ± 6	8 ± 5	10 ± 5	0.51
AHI (events/h)	50 ± 19	54 ± 21	48 ± 20	48 ± 17	0.757
ODI (events/h)	43 ± 18	45 ± 22	40 ± 17	43 ± 17	0.79
TST90 (%)	14 ± 14	14 ± 12	17 ± 17	11 ± 11	0.962
Minimal SpO_2_ (%)	77 ± 8	77 ± 10	77 ± 6	78 ± 7	0.109
Baseline SpO_2_ (%)	93 ± 1	94 ± 1	93 ± 2	93 ± 1	0.972

*Note*: Data are expressed as mean ± SD or *n* (%). For detailed description of the baseline characteristics of the cohort, please see (O'Donnell et al. 2024).

Abbreviations: AHI = apnoea/hypopnoea index, BMI = body mass index, HDL = high‐density lipoproteins, LDL = low‐density lipoproteins, ODI = oxygen desaturation index, SpO_2_ = oxygen saturation, TST90 = percent of oxygen saturation of total sleep time below 90%.

We next investigated the effects of our interventions on body weight and other anthropometric parameters (Table 3). After 24 weeks of treatment, subjects in the Liraglutide and combination groups lost 6.17 ± 3.57 kg (−5.7%, *p* < 0.001) and 3.67 ± 4.22 kg (−3.5%, *p* = 0.031), respectively, with a proportional decrease in waist size and BMI. In contrast, those on CPAP alone demonstrated a small weight gain (2.73 ± 4.58 kg, 2.5%, *p* = 0.092). To gain a detailed insight into the effects of the interventions on AT compartments, we measured visceral and subcutaneous AT depot volumes on the CT component of the 18F‐FDG PET‐CTs performed at baseline and 24 weeks. Weight loss driven by Liraglutide reduced both compartments in proportion (Table 3), with a 12% and 14% reduction in visceral AT in those patients taking Liraglutide alone or combination therapy, respectively. In the whole population, change in visceral but not subcutaneous fat mass correlated significantly with change in oxygen desaturation index (ODI) as a marker of IH severity (*r* = 0.379, *p* = 0.048) but there was no relationship with AHI.

**TABLE 3:** Anthropometric parameters, adipose tissue volumes and glycaemic parameters at baseline and after 24 weeks of treatment.

	Liraglutide		CPAP		Combination	
	Pre	24 weeks	Pre	24 weeks	Pre	24 weeks
Weight (kg)	107 ± 14	**101 ± 13** *	108 ± 10	111 ± 10	104 ± 13	**100 ± 13** *
BMI (kg/m^2^)	35.14 ± 3.02	**33.11 ± 3.12** *	35.18 ± 3.59	36.04 ± 3.29	34.41 ± 3.23	**33.16 ± 3.29** *
Waist circumference (cm)	119 ± 9	115 ± 9	122 ± 10	123 ± 9	117 ± 11	111 ± 10
Waist:hip ratio	1.02 ± 0.87	1.02 ± 0.06	1.08 ± 0.08	1.07 ± 0.12	1.04 ± 0.05	0.99 ± 0.06
Total AT volume (cm^3^)	105.8 ± 27.9	**91.9 ± 26.3**	93.5 ± 16.8	**96.7 ± 16.3**	97.7 ± 18.5	**86.0 ± 22.5**
Subcutaneous AT volume (cm^3^)	63.5 ± 22.2	**55.8 ± 21.5**	53.7 ± 15.3	54.8 ± 14.3	61.5 ± 18.4	**54.7 ± 17.9**
Visceral AT volume (cm^3^)	42.2 ± 13.5	**36.1 ± 10.0**	39.8 ± 8.0	42 ± 8.4	36.2 ± 7.6	**31.2 ± 9.0**
HbA1c (mmol/mol)	38.3 ± 3.9	36.3 ± 5.4*	37.6 ± 2.8	**38.9 ± 3.2**	38.5 ± 2.2	**35.2 ± 3.6** *
Fasting glucose (mmol/L)	5.4 ± 0.4	**5.0 ± 0.5**	5.3 ± 0.5	5.1 ± 1.1	5.7 ± 0.6	**5.2 ± 0.3**
Post‐prandial glucose (mmol/L)	7.76 ± 1.90	**6.0 ± 2.5**	7.5 ± 2.7	**6.0 ± 1.5**	8.0 ± 3.7	**5.9 ± 2.7**

*Note*: Values indicate mean ± SD. ‘Bold’ indicates *p* < 0.05 for post‐treatment value as compared to baseline value. Bright green indicates ‘most beneficial’, light green indicates ‘intermediate benefit’ and gold indicates ‘neutral/negative effect’.

Abbreviations: AT = adipose tissue, BMI = body mass index.

* *p* < 0.05 versus CPAP alone.

### 3.5 Liraglutide‐Mediated Weight Loss but Not CPAP Therapy Improves Insulin Sensitivity in OSA Subjects

**FIGURE 6:**
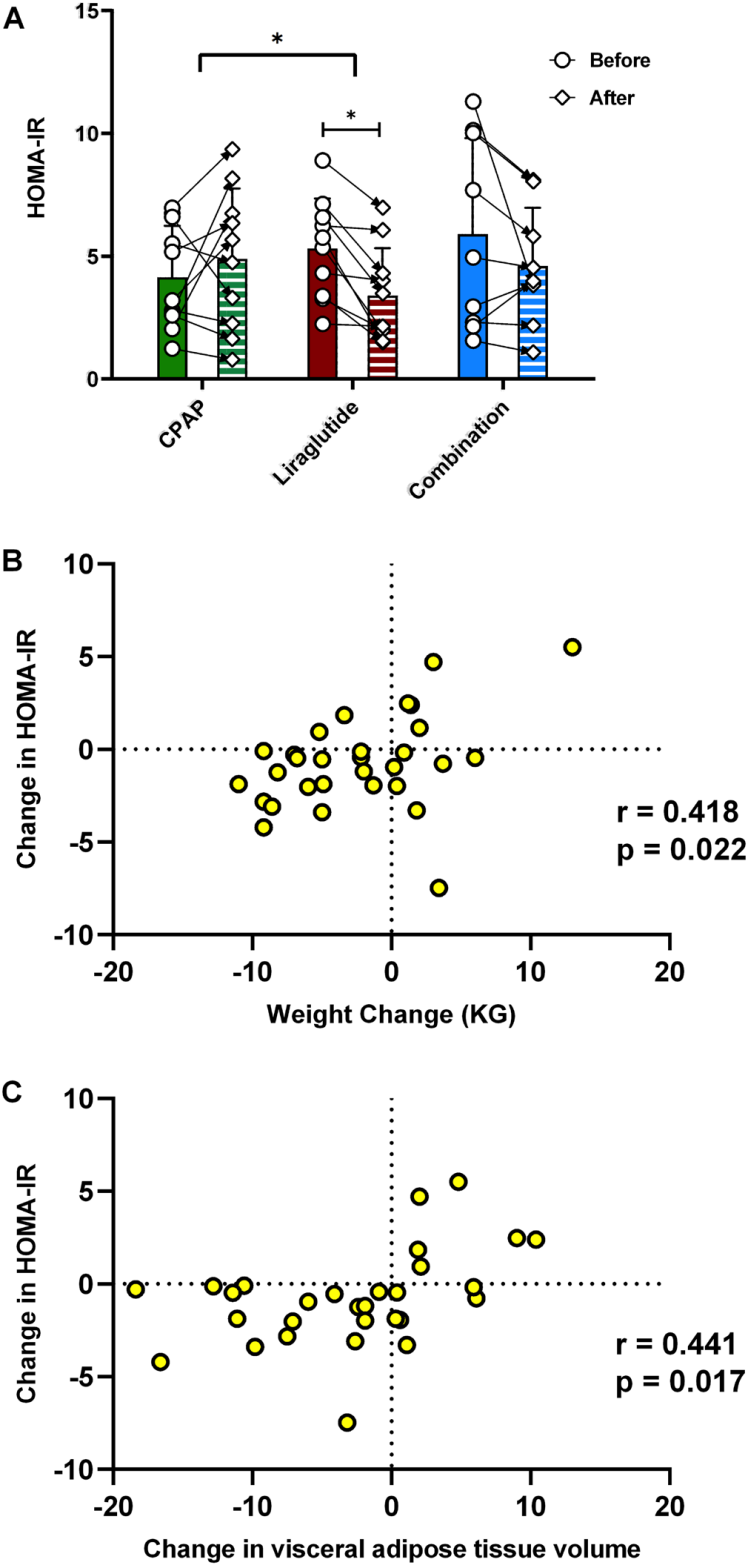
Liraglutide but not CPAP leads to improvement in insulin resistance in OSA subjects. HOMA‐IR values at baseline and after 24 weeks of respective treatments (A). Data represent mean ± SD and individual point‐to‐point changes, *p < 0.05. (B) Scatterplot of change in weight versus change in HOMA‐IR and (C) scatterplot of change in visceral adipose tissue volume versus change in HOMA‐IR.

We next aimed to test the effect of the different treatments on insulin sensitivity using HOMA‐IR as a surrogate marker, and this was the primary aim of the RCT. At baseline, there was a high degree of IR present, with an overall HOMA‐IR of 5.25 ± 2.75, and there were no significant differences between groups. After 24 weeks of treatment, HOMA‐IR reduced in the Liraglutide group and trended towards reduction in the combination group (from 5.32 ± 2.03 to 3.40 ± 1.93, *p* = 0.002 and from 5.89 ± 3.92 to 4.60 ± 2.38, *p* = 0.186, respectively), with no improvement in the CPAP group (Figure 6A). Change in HOMA‐IR correlated significantly with weight change, with those who lost the most weight having the greatest improvement (Figure 6B). This appeared to be predominantly driven by reduction in visceral rather than subcutaneous fat, with a strong correlation between the reduction in visceral AT and the improvement in HOMA‐IR (*r* = 0.441, *p* = 0.017) (Figure 6C). Furthermore, using a linear regression analysis including baseline demographic and anthropometric parameters and change of BMI and fat depots as variables, only change in visceral fat mass remained an independent predictor of change in insulin sensitivity (regression coefficient *β* = 0.441, *p* = 0.017). Furthermore, there was no relationship between any change in OSA parameters and HOMA‐IR.

Thus, Liraglutide‐mediated reduction in visceral AT volume improves insulin sensitivity.

### 3.6 Combination Therapy With Liraglutide and CPAP Leads to Greater Improvement in Fasting Glucose, Glucose Tolerance and HbA1c Than Either Treatment Alone

Apart from investigating the effect on insulin sensitivity, we also evaluated the potential influence of the different interventions on fasting glucose, glucose tolerance, and HbA1c. At baseline, 14 patients (47%) (6 CPAP, 4 Liraglutide, 4 combination) met the diagnostic criteria for pre‐diabetes as defined by a fasting blood glucose of 5.6–6.9 mmol/L, and/or a 2‐h postprandial glucose of 7.8–11 mmol/L, or a HbA1c between 39 and 47 mmol/mol. Liraglutide alone improved fasting glucose and glucose tolerance. The latter was also improved by CPAP alone, but interestingly, the greatest improvement in all parameters was seen with combination treatment, although changes were not statistically significant given the low number of subjects (Table 3). With the changes in fasting and postprandial glucose, resolution of pre‐diabetes status was seen in one patient in the CPAP group, four patients in the Liraglutide group and two patients in the combination group.

Thus, CPAP alone improves glucose tolerance, but combination with Liraglutide appears to provide the greatest benefit.

## 4 Discussion

In this report, we show that Liraglutide‐mediated weight loss is an effective treatment strategy to improve insulin sensitivity in OSA subjects. However, as demonstrated by our extensive translational approach, Liraglutide is unable to abolish the perturbations in glucose metabolism which are specifically triggered by IH and weight loss in combination with CPAP appears most beneficial.

Despite its limitations, mainly due to the explorative nature of the RCT, our study is an important contribution to the ongoing debate of the role of GLP‐1 analogues in the management of OSA. Sparked, appropriately, by the Surmount‐OSA trial (Malhotra et al. 2024), there is increasing optimism for having identified an effective treatment alternative to the, for many subjects, cumbersome CPAP therapy. At the time of our studies, Liraglutide was the only approved GLP‐1 pharmacotherapy for obesity management, and one may argue that the newer generation GLP‐1‐based agents such as Tirzepatide facilitate greater weight loss and hence may have led to superior outcomes. While the definitive answer to this hypothesis is outstanding, we certainly need to advise caution. Although beneficial in reducing AHI, even bariatric surgery, as the most effective weight loss strategy, will frequently not lead to remission of OSA (Al Oweidat et al. 2023; Wong et al. 2018) and OSA subjects are at higher risk for weight re‐gain than non‐OSA counterparts following this intervention (Dalmar et al. 2018; Messineo et al. 2024). With regards to GLP‐1 analogues, the Surmount‐OSA trial reported a mean reduction in AHI of 25 events/h over 52 weeks with Tirzepatide, which still leaves a substantial proportion of patients with likely clinically significant disease. Unfortunately, discontinuation of the treatment also leads to rapid re‐gain of weight (Aronne et al. 2024) and as of now, uncertainty persists in relation to long‐term adherence, efficiency, and side effects of GLP‐1‐based medications in real‐world populations. Moreover, we are still lacking data on the usefulness of those agents on patient‐related outcomes such as daytime sleepiness or quality of life and especially on long‐term cardiometabolic outcomes.

Our study emphasises that with the continuation of IH as the primary trigger of the adverse metabolic responses through the promotion of a pro‐inflammatory phenotype of the visceral AT, a complete elimination of those negative effects with weight loss alone is perhaps not to be expected. Furthermore, IH‐induced metabolic perturbations may also simply not be completely reversible. This hypothesis has been promoted by several murine studies using a protocol where IH exposure was followed by a period of normoxic recovery, and the latter failed to result in reversal of the adverse consequences (Gileles‐Hillel et al. 2017; Polak et al. 2013). Although speculative at this stage, IH may also act through pathways that are not collectively accessible by GLP‐1 analogues. Our cell culture results are in support of this position, demonstrating that Liraglutide, although effective at the RNA level in mitigating pro‐inflammatory macrophage polarisation, did not result in significant attenuation of pro‐inflammatory cytokine secretion.

Our study has significant strengths, including its comprehensive translational nature, ranging from a state‐of‐the‐art in vitro model of IH over an extensively validated murine model of IH to a proof‐of‐concept study of a carefully selected cohort of OSA subjects. It is the first to investigate the effects of GLP‐1 analogues on IH‐mediated metabolic complications, and our results have important clinical implications, highlighting the need for holistic, personalised treatment approaches and careful monitoring for metabolic health.

Apart from the already mentioned limitation of the human study, several others have also to be acknowledged. First, there are the inevitable limitations of the preclinical models of IH which have already been extensively discussed (Farre et al. 2022). Furthermore, our investigation both in mice and humans focused on IH in the presence of obesity which comprises the majority of the OSA population. However, as a consequence, our results cannot be extrapolated to lean conditions or subjects with Class 3 obesity where effects on body composition may significantly outweigh fluctuations of the degree of sleep‐disordered breathing. In addition, we excluded subjects with established diabetes to avoid confounding effects of GLP‐1 on hyperglycaemia in this patient group. Moreover, a potential added effect of either treatment beyond the chosen duration of 24 weeks cannot be excluded. Noteworthy, it was not within the scope of this study to explain the detailed mechanisms of the limited benefit of Liraglutide on IH‐mediated metabolic function. Specifically targeted translational studies, however, need to urgently address this point.

In conclusion, our study provides strong evidence that neither weight loss without considerable impact on IH nor effective suppression of sleep‐disordered breathing alone is effective in restoring glycaemic health in OSA. It highlights the urgent need for further detailed mechanistic studies on the association of IH and metabolic diseases and for large, well‐designed randomised‐controlled trials exploring the benefit of combined treatment approaches and to define the clinical phenotypes which respond best to a given strategy employing precision medicine. Management of obesity requires to be embedded in a holistic management strategy of OSA. However, the exact place of GLP‐1 analogues remains to be defined.

## Author Contributions

**Cliona O'Donnell:** investigation, formal analysis, project administration, writing – original draft, writing – review and editing. **Ailbhe King:** investigation, formal analysis, writing – original draft, writing – review and editing. **Guillaume Vial:** investigation, formal analysis, writing – review and editing. **Emily O'Neill:** investigation, formal analysis, writing – review and editing. **Shane Crilly:** investigation, formal analysis, writing – review and editing. **Jonathan D. Dodd:** methodology, investigation, validation, formal analysis, supervision, writing – review and editing. **David J. Murphy:** methodology, investigation, validation, formal analysis, supervision, writing – review and editing. **Elise Belaidi:** investigation, formal analysis, supervision, writing – review and editing. **Jean‐Louis Pepin:** investigation, formal analysis, supervision, resources, writing – review and editing. **Claire Arnaud:** investigation, methodology, formal analysis, supervision, writing – review and editing. **Donal O'Shea:** investigation, formal analysis, writing – review and editing. **Silke Ryan:** conceptualization, methodology, investigation, validation, formal analysis, supervision, funding acquisition, project administration, resources, writing – original draft, writing – review and editing.

## Conflicts of Interest

J.D.D. received royalties from Amirsys Inc. for authorship, J.‐L.P. received consulting fees from ResMed, Philips, SEFAM, Bioprojet, Idorsia, Zoll and Pharmanovia, S.R. received unrestricted grants from NovoNordisk and Fitbit, consulting fees for the Irish Rugby Football Union and lecture honoraria from the American College of Cardiology and the Chinese Thoracic Society. The other authors declare no conflicts of interest.

## Data Availability

The data that support the findings of this study are available on request from the corresponding author. The data are not publicly available due to privacy or ethical restrictions.
